# Evaluation of the Reconstruction Parameters of Brain Dopamine Transporter SPECT images Obtained by a Fan Beam Collimator: A Comparison with Parallel-hole Collimators

**DOI:** 10.22038/aojnmb.2018.10330

**Published:** 2018

**Authors:** Keishin Morita, Akira Maebatake, Rina Iwasaki, Yuki Shiotsuki, Kazuhiko Himuro, Shingo Baba, Masayuki Sasaki

**Affiliations:** 1 Division of Medical Quantum Science, Department of Health Sciences, Graduate School of Medical Sciences, Kyushu University, Fukuoka, Japan; 2 Radiological Science Course, Department of Health Sciences, School of Medicine, Kyushu University, Fukuoka, Japan; 3 Division of Radiology, Department of Medical Technology, Kyushu University Hospital, Fukuoka, Japan; 4 Department of Clinical Radiology, Graduate School of Medical Sciences, Kyushu University, Fukuoka, Japan

**Keywords:** Dopamine transporter, Fan beam collimator, SPECT/CT

## Abstract

**Objective(s):**

The purpose of this study was to examine the optimal reconstruction parameters for brain dopamine transporter SPECT images obtained with a fan beam collimator and compare the results with those obtained by using parallel-hole collimators.

**Methods:**

Data acquisition was performed using two SPECT/CT devices, namely a Symbia T6 and an Infinia Hawkeye 4 (device A and B) equipped with fan-beam (camera A-1 and B-1), low- and medium-energy general-purpose (camera A-2 and B-2), and low-energy high-resolution (camera A-3 and B-3) collimators. The SPECT images were reconstructed using filtered back projection (FBP) with Chang’s attenuation correction. However, the scatter correction was not performed. A pool phantom and a three-dimensional (3D) brain phantom were filled with ^123^I solution to examine the reconstruction parameters. The optimal attenuation coefficient was based on the visual assessment of the profile curve, coefficient of variation (CV) [%], and summed difference from the reference activity of the pool phantom. The optimal Butterworth filter for the 3D-brain phantom was also determined based on a visual assessment. The anthropomorphic striatal phantom was filled with ^123^I solution at striatum-to-background radioactivity ratios of 8, 6, 4, and 3. The specific binding ratio (SBR) of the striatum (calculated by the CT method) was used to compare the results with those of the parallel-hole collimators.

**Results:**

The optimal attenuation coefficients were 0.09, 0.11, 0.05, 0.05, 0.11, and, 0.10 cm^-1^ for cameras A-1, A-2, A-3, B-1, B-2, and B-3, respectively. The cutoff frequencies of the Butterworth filter were 0.32, 0.40, and 0.36 cycles/cm for camera A, and 0.46, 0.44, and 0.44 cycles/cm for camera B, respectively. The recovery rates of the SBR_mean_ with camera A were 51.2%, 49.4%, and 45.6%, respectively. The difference was not statistically significant. The recovery rates of the SBR with camera B were 59.2%, 50.7%, and 50.8%, respectively. Camera B-1 showed significantly high SBR values.

**Conclusion:**

As the findings indicated, the optimal reconstruction parameters differed according to the devices and collimators. The fan beam collimator was found to provide promising results with each device.

## Introduction

Some movement disorders are associated with the loss of dopaminergic neurons. Dopamine transporter (DaT) is located on the dopaminergic nerve terminals in the nigrostriatal pathway. DaT exists in the striatum, which is a component of the basal ganglia, including putamen and caudate nucleus. DaT single-photon emission computed tomography (SPECT) using ^123^I-FP-CIT (ioflupane) is an important tool for the diagnosis of neurologic disorders (1, 2). DaT SPECT has been reported to be useful for diagnosing Parkinson’s disease, parkinsonian syndrome, and dementia with Lewy bodies (3-6).

Radiotracer neuroimaging techniques using positron emission tomography (PET) or SPECT can be helpful in visualizing and measuring striatal dopaminergic activity (7, 8). PET scan provides higher spatial resolution and sensitivity in comparison to the SPECT scan. However, previous studies demonstrated that the striatal uptakes of ^123^I-FP-CIT SPECT and ^18^F-DOPA PET correlate well with each other in the patients with different stages of Parkinson’s disease (9, 10).

^123^I-FP-CIT might be more sensitive than ^18^F-DOPA in detecting the early striatal dopaminergic deficits because DaT is downregulated as an early response to the decrease in the amount of endogenous dopamine (11).

The DaT SPECT is performed by means of a parallel-hole collimator in the majority of the institutions. The guidelines for DaT SPECT recommend the use of low-energy high-resolution (LEHR) parallel-hole collimator because the striatum is a small structure (1, 2). However, our previous studies revealed a difference in the quantitative accuracy of the LEHR collimators produced by different manufacturers (12, 13). A fan beam collimator is expected to provide better results in terms of the spatial resolution than parallel-hole collimators. The cerebral blood flow SPECT is often performed by using a fan beam collimator (14-16). However, imaging of DaT SPECT using a fan beam collimator has not been sufficiently investigated yet.

The purpose of this study was to examine the optimal reconstruction parameters for brain dopamine transporter SPECT imaging with a fan beam collimator and compare the results with those obtained using parallel-hole collimators.

## Methods

***Phantoms***

A pool phantom with a 16 cm diameter, 15 cm height, and 3,016 mL volume (Akita Machine-engineering, Japan) was used to determine the attenuation coefficient of Chang’s method (12). The phantom was filled with 14.7 kBq/mL of^ 123^I solution. The three-dimensional (3D) brain phantom (Molecular Imaging Labo Inc., Osaka, Japan) was used to determine the cutoff frequency of the Butterworth filter (12, 17). The 3D brain phantom was made of a transparent photocurable polymer or polyepoxide (density, 1.07 g/mL) and constructed with a laser modeling technique. The structure precisely imitated the gray matter, white matter, cerebrospinal fluid space, skull, and scalp based on the magnetic resonance (MR) images. The bone region was filled with K_2_HPO_4_ (310.3 mL). The gray matter region (562.2 mL) was filled with a ^123^I solution (radioactivity, 29.6 kBq/mL).

**Table 1:** The SPECT/CT protocol

**Device and collimator**	**Camera A-1**	**Cameras A-2 and A-3**	**Camera B-1**	**Cameras B-2 and B-3**
Energy window	159 keV±10%		159 keV±10%	
Acquisition matrix	256×256	128×128	128×128	
Zoom	1.23	1.45	1.00	1.34
Pixel size	1.95	3.30	1.93	3.30
Acquisition angle	3°	4°	3°	4°
Acquisition time	5 min×6 times		5 min×6 times	
Image reconstruction	FBP (ramp)		FBP (ramp)	
Preprocessing filter	Butterworth (order: 8)		Butterworth (order: 8)	
Attenuation correction	Chang		Chang	
Scatter correction	-		-	

FBP: filter back projection

**Table 2:** The target and actual SBR_true _values

**Camera**	**Target SBR**_true _ **(S/B ratio)**			
	**7** **(8)**	**5** **(6)**	**3** **(4)**	**2** **(3)**
Camera A-1	6.99	4.50	3.24	1.93
Camera B-1	6.66	4.79	2.84	1.90

**Table 3.** The optimal reconstruction parameters for each collimator

**Device & collimator**	**μ value (cm** ^-1^ **)**	**Butterworth filter (cycles/cm)**	
		**order**	**Cut-off**
Camera A-1	0.09	8	0.32
Camera A-2	0.11	8	0.40
Camera A-3	0.05	8	0.36
Camera B-1	0.05	8	0.46
Camera B-2	0.11	8	0.44
Camera B-3	0.10	8	0.44

**Table 4:** The spatial resolution of FWHM for each collimator

**Collimator**	**Axial (mm)**	**Transaxial (mm)**
Camera A-1	11.4	11.2
Camera A-2	16.7	16.3
Camera A-3	15.8	15.7
Camera B-1	12.0	12.3
Camera B-2	16.2	16.2
Camera B-3	14.0	14.1

**Table 5:** The recovery and linearity of SBR_SPECT_

	**Recovery of SBR**_SPECT_						**Linearity** **(R** ^2^ **)**
	** SBR**_true_					**Average**	
	**Target**	**2**	**3**	**5**	**7**		
	**Actual for camera A**	**1.93**	**3.24**	**4.50**	**6.99**		
	**Actual for camera B**	**1.90**	**2.84**	**4.79**	**6.66**		
SBR_mean_							
Camera A-1		47.3%	54.9%	51.1%	51.4%	51.2±3.1%	0.99
Camera A-2		50.8%	46.2%	50.9%	49.9%	49.4±2.2%	1.00
Camera A-3		51.2%	43.2%	42.5%	45.2%	45.6±4.0%	0.99
Camera B-1		59.0%	60.2%	58.5%	59.2%	59.2±0.7%	1.00
Camera B-2		55.7%	51.2%	50.3%	48.5%	50.7±1.8%**	1.00
Camera B-3		55.2%	46.5%	52.9%	48.7%	50.8±3.9%**	0.98
SBR_max_							
Camera A-1		77.9%	77.4%	87.0%	69.2%	77.9±7.3%	0.95
Camera A-2		74.6%	60.7%	67.8%	64.7%	67.0±5.9%	0.99
Camera A-3		72.1%	61.1%	57.9%	64.6%	63.9±6.1%*	0.98
Camera B-1		81.6%	79.6%	83.3%	85.6%	82.5±2.6%	1.00
Camera B-2		75.1%	66.0%	68.6%	68.2%	69.5±3.9%**	1.00
Camera B-3		78.1%	70.3%	75.2%	65.8%	72.3±5.4%**	0.98

* *P*<0.05 (vs. camera A-1)

** *P*<0.05 (vs. camera B-1)

**Figure 1:**
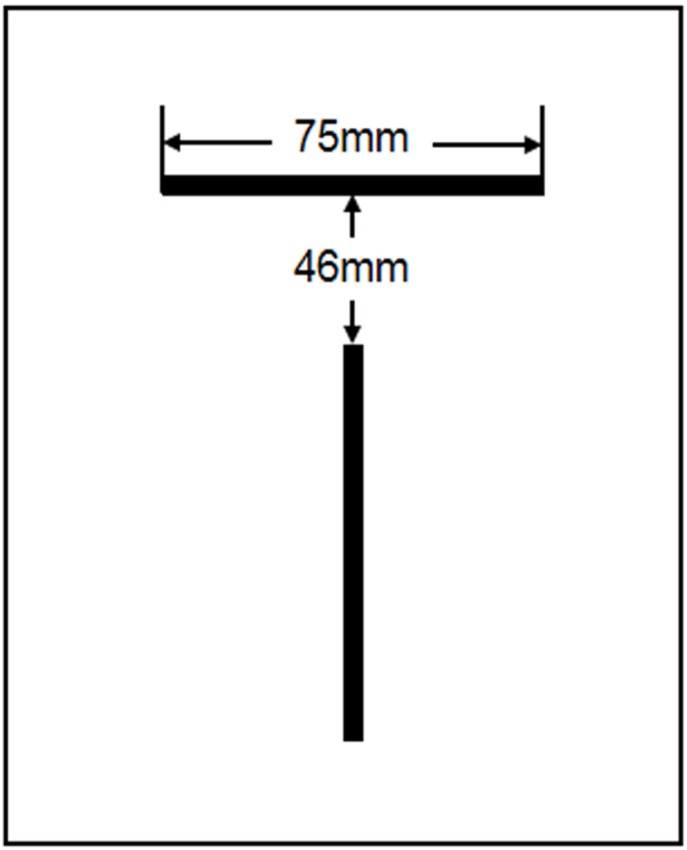
The line sources in a single plane

**Figure 2:**
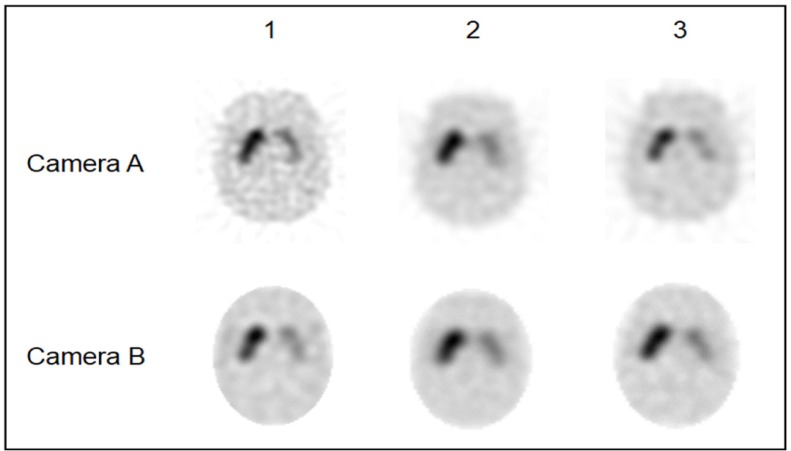
SPECT images reconstructed using the optimal reconstruction parameters (The images in the upper row were obtained using camera A, and the images in the lower row were achieved using camera B. The striatum was clearly observed on images using cameras A-1 and B-1.)

**Figure 3:**
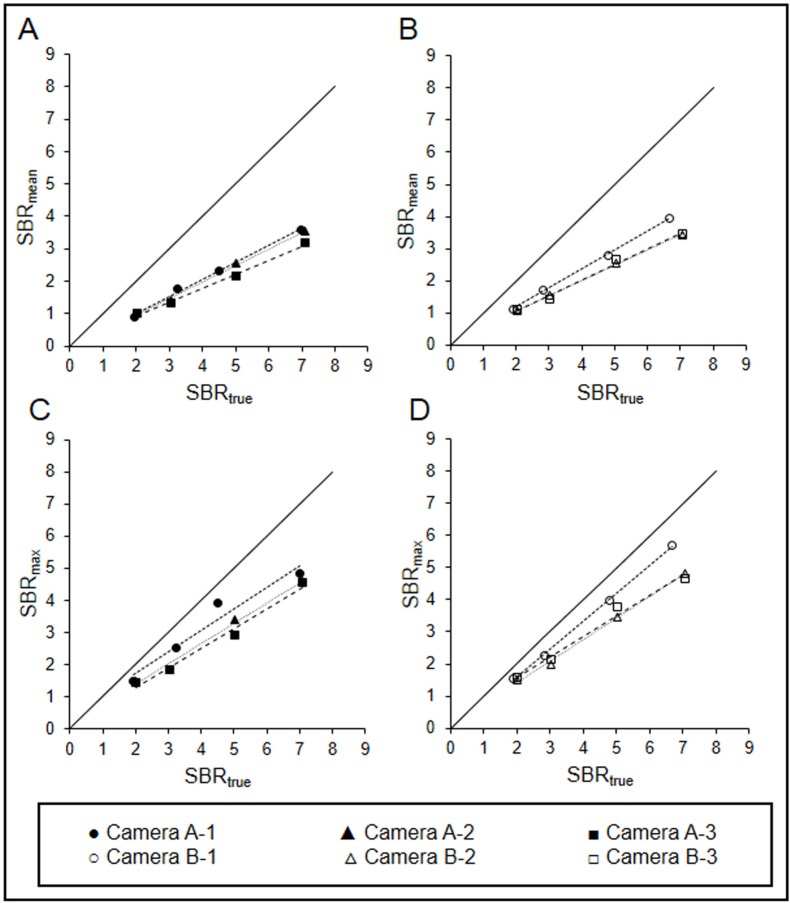
Correlation between SBR_true_ and SBR_SPECT_ of SBR_mean _(A, B) and SBR_max _(C, D), (A, C) Symbia T6, (B, D) Infinia Hawkeye 4 (Good linearity was observed for all collimators

The line source with ^123^I was used to evaluate the spatial resolution. A glass capillary (with internal diameter of 0.426 mm and length of 75 mm) was filled with ^123^I solution (radioactivity, 111 MBq/mL). Two line sources were located 46 mm apart on an orthogonal position in a single plane (Figure 1). The evaluation of DaT SPECT images was performed using an anthropomorphic striatal phantom (NMP Business Support Co. Ltd., Tokyo, Japan) (12, 13). 

The phantom consisted of chambers for the right and left striatum (12.5 mL) and the cerebrum (1,180 mL) based on the MR images of a healthy subject. The phantom was filled with a different ^123^I solution with the radioactivity values of 44.4 and 22.2 kBq/mL for the right and left striatum, respectively. The background values were 5.55 and 7.43 kBq/mL for the right and left striatum, respectively. Four striatum-to-background radioactivity ratios (S/B ratios) (i.e., 8, 6, 4, and 3) were examined in this study.

***Imaging protocol***

The data were acquired using two SPECT/CT devices. Symbia T6 (Siemens Healthcare, Erlangen, Germany) was equipped with a low-to-medium energy fan beam (LMEFB) collimator (camera A-1), a low-to-medium energy general-purpose (LMEGP) collimator (camera A-2), and an LEHR collimator (camera A-3). Furthermore, Infinia Hawkeye 4 (GE Healthcare, Buckinghamshire, UK) was equipped with a low-energy fan beam (FAN) collimator (camera B-1), an extended low-energy general-purpose (ELEGP) collimator (camera B-2), and an LEHR collimator (camera B-3).

The data acquisition was performed six times in continuous mode with clockwise and counterclockwise rotations for 5 min per 180°. The matrix sizes were 256×256 and 128×128, and the pixel sizes were 1.95×1.95 and 1.93×1.93 mm (1.23 zoom for camera A-1 and 1.00 zoom for camera B-1), respectively. The matrix and pixel sizes of the parallel-hole collimator were determined in our previous study (12). 

The attenuation correction was performed using Chang’s method; however, the scatter correction was not performed. The SPECT/CT protocols for each device and collimator are summarized in Table 1. Image reconstruction was performed on syngo MI Applications (Siemens Healthcare, Erlangen, Germany) and Xeleris 2 devices (GE Healthcare, Buckinghamshire, UK).

***Reconstruction parameters***

The SPECT images were reconstructed using the filtered back projection (FBP) with a ramp filter. The attenuation coefficient for Chang’s method was determined by a pool phantom examination in accordance with the method described in our previous study (12). At that time, a Butterworth filter was used for smoothing (order of 8 and cutoff frequencies of 0.44 cycles/cm for cameras A-1, B-2, and B-3, 0.40 cycles/cm for camera A-2, 0.36 cycles/cm for camera A-3 and 0.46 cycles/cm for camera B-1). 

The μ value of the attenuation coefficient varied from 0.01 to 0.15 at 0.01 intervals. The evaluations of the pool phantom images included the visual assessment of the flatness of the profile curve, coefficient of variance (CV), and summed difference from the reference activity (12, 13). The flatness of the profile curve was visually classified into five grades by five nuclear physicians (i.e., -2: obviously concave, -1: probably concave, 0: flat, +1: probably convex and +2: obviously convex).

The CV was calculated by dividing the percentage of standard deviation by the mean activity of a circular region of interest (ROI) (diameter: 16 cm) on the phantom image. To calculate the summed difference from the reference activity, a rectangular ROI (30×35 pixel) was placed on the reconstruction image, and the mean activity of the two top corner pixels was used as a reference value. Subsequently, the difference between the reference activity and each pixel value in the ROI was summed up (positive: convex, negative: concave). Finally, the μ values were determined by the comprehensive evaluation of these results.

The cutoff frequency of the Butterworth filter, which was determined based on the visual assessment of the SPECT images of the 3D brain phantom, ranged within 0.18-0.62 at 0.02 intervals. The optimal cutoff frequencies of the Butterworth filter for parallel-hole collimators were determined in our previous study (12). At that time, the attenuation coefficient was determined by the abovementioned method, and the order of 8 was used for the Butterworth filter. Five nuclear medicine physicians visually classified the SPECT image using a rating scale ranging within -2 to +2 (i.e., -2: extremely poor, -1: poor, 0: normal, +1: good and +2: excellent).

***Spatial resolution***

Spatial resolution was evaluated using the SPECT images of line sources. The full width at half maximum (FWHM) was measured on a slice with the maximum count of a phantom using Prominence Processor, Version 3.1 (Nihon Med-Physics Corp., Tokyo, Japan). The FWHM included the axial FWHM calculated by averaging the X and Y axis FWHM and the trans-axial FWHM of the Z axis.

***Specific binding ratio***

The ROI of the striatum was the contour of each striatum (determined using the CT image), which was copied onto the SPECT image on the slice with the maximum count for the striatum. For the background, a 10×10 pixel rectangular ROI was placed on the occipital region. The ROI was placed manually and individually by the consensus of a nuclear medicine physician and a radiologic technologist. The specific binding ratio (SBR) was calculated as follows:

$\mathrm{SBR}=\frac{C_{s}-C_{b}}{C_{b}}$

where C_s_ is the count of the striatum, and C_b_ is the mean count of the background area. 

The SBR using the mean SPECT count of the striatum (C_s, mean_) is referred to as SBR_mean_, while the SBR using the maximum SPECT count of the striatum (C_s, max_) is referred to as SBR_max_. The SBR_SPECT_ consists of both SBR_mean _and SBR_max _values. The true SBR (SBR_true_) was calculated using the radioactivity of the ^123^I solution in the striatum and background, which was measured using an automatic well γ counter (AccuFLEX γ7001, Hitachi Aloka Medical, Ltd.) and considered as a reference value (Table 2). 

When the scan was initiated, the S/B ratios for camera A-1 were 7.99, 5.50, 4.24, and 2.93, respectively; therefore, the SBR_true_ values were 6.99, 4.50, 3.24, and 1.93, respectively. Furthermore, the S/B ratios for camera B-1 were 7.66, 5.79, 3.84, and 2.90, respectively; consequently, the SBR_true_ values were 6.66, 4.79, 2.84, and 1.90, respectively.

***Statistical analysis***

The SBR_SPECT_ was evaluated by comparing its recovery and linearity with those of the SBR_true_. The difference in the recovery of SBR_SPECT_ was analyzed by the Tukey-Kramer test. The linearity was analyzed by calculating the correlation coefficient between SBR_SPECT_ and SBR_true_. P-value less than 0.05 was considered statistically significant.

## Results

***Determination of reconstruction parameters***

The optimal μ values for Chang’s attenuation correction were 0.09 cm^-1^ for camera A-1, 0.11 cm^-1^ for cameras A-2 and B-2, 0.05 cm^-1^ for cameras A-3 and B-1, and 0.10 cm^-1^ for camera B-3. The most appropriate cutoff frequencies of the Butterworth filter were 0.32 and 0.46 cycles/cm for cameras A-1 and B-1, respectively. The optimal reconstruction parameters determined for each device and collimator are displayed in Table 3. Table 3 also includes the parameters for parallel-hole collimators, which were indicated in our previous study (12).

***Spatial resolution***

The axial and trans-axial spatial resolutions of the cameras are shown in Table 4. The fan beam collimators of both devices (i.e., cameras A-1 and B-1) showed better spatial resolution in comparison to the parallel-hole collimators. The FWHM of camera A-1 showed the best spatial resolution (axial: 11.4 mm, trans-axial: 11.2 mm).

***Comparison of DaT SPECT among different devices and collimators***

Figure 2 shows the SPECT images reconstructed using the optimal reconstruction parameters. The striatum was more clearly observed on the images obtained by fan beam collimators (cameras A-1 and B-1) in comparison to images obtained using parallel-hole collimators (cameras A-2, A-3, B-2, and B-3). The recovery and linearity of SBR_SPECT_ were compared among the cameras (Table 5 and Figure 3). 

For cameras A, camera A-1 showed the highest SBR_mean_, followed by cameras A-2 and A-3. However, the difference was not statistically significant. Camera A showed the highest recovery of SBR_max_. The recovery of SBR_max_ of camera A-1 (77.9 %) was significantly higher than that of camera A-3 (63.9 %) (*P*<0.05). In contrast, the highest recovery rates of SBR_mean _and SBR_max _for camera B and camera B-1 were 59.2% and 82.5%, respectively. The recovery of SBR_mean _and SBR_max_ of camera B-1 was significantly higher than those of cameras B-2 and B-3 (*P*<0.05). Good linearity was observed for all collimators.

## Discussion

In this study, we determined the optimal reconstruction parameters for DaT SPECT images obtained with a fan beam collimator, and compared the results to those obtained using parallel-hole collimators. The optimal μ values for cameras A-1 and B-1 were 0.09 and 0.05 cm^-1^, respectively. Furthermore, the most appropriate cutoff frequencies of the Butterworth filter were 0.32 and 0.46 cycles/cm for cameras A-1 and B-1, respectively. The comparison of different devices and collimators revealed that the devices using a fan beam collimator achieved better results than those utilizing a parallel-hole collimator.

Our results revealed that the best recovery of SBR_mean_ and SBR_max_ was obtained with a fan beam collimator. The fan beam collimator provides greater spatial resolution than parallel-hole collimators. This fact was also indicated by the FWHM results obtained by using line sources. Furthermore, Camera A-1 is designed for low- and medium-energy photons. A medium energy collimator reduces the septal penetration of the high-energy ɤ rays emitted by ^123^I (18). On the other hand, camera B-1 is designed for the low-energy photons. 

Our results also showed that the µ values of cameras A-3 and B-1 were lower than those of the others cameras. These cameras are basically designed for ^99m^Tc; therefore, the septal penetration of the high-energy ɤ rays could not be eliminated. The scatter correction could not be performed in the present study; thus, the scatter photons, including penetration, were considered to lead to image deterioration. The septal penetration of ^123^I high-energy photons is considered to reduce the signal-to-noise ratio and resolution (19, 20). This may have resulted in obtaining lower spatial resolution for camera B-1, compared to that of camera A-1. Therefore, the trade-off between spatial resolution and septal penetration should be considered when deciding which collimation to use (19).

Although the relationship of FWHM with the recovery of SRB_SPECT_ reflected the characteristics of the collimators in each device, it did not reflect, when compared between cameras. For example, cameras A-1 and B-1 showed opposite results regarding the FWHM and recovery of SRB_SPECT_. The cause of this fact has not been well clarified; however, the different characteristics of the devices, such as the system sensitivity and reconstruction algorithms, may influence the results. Therefore, further examination is required to solve this issue.

In the present study, the ROI for the striatum was manually placed along the contours of the striatum on a CT image. The radioactivity may be underestimated in small structures, such as striatum, because of the partial volume effect (PVE) (21, 22). Buvat et al. reported that the radioactivity concentration of the striatum was underestimated by more than 50% without partial volume correction (23). Moreover, in another study, the partial volume correction was particularly effective in reducing the underestimation of the putaminal uptake (24). Bolt et al. used a pentagonal ROI for the striatum, including striatal counts with spill out activity around the striatum (25). They successfully excluded the influence of the PVE to obtain the SBR. When the ROI is placed manually, the inter-observer variation is considered to influence the reliability of the SBR. The inter- and intra-observer variation of the results should be examined in the future studies. Furthermore, the template ROI is considered to reduce both the inter- and intra-observer variabilities.

This study compared the SBR_SPECT_ between a fan beam collimator and a parallel-hole collimator without scatter correction. Since scatter correction could not be performed for camera B-1, we did not perform it for any cameras. Therefore, scatter correction was not performed to reduce its effect. Cot et al. indicated that scatter correction progressively improved the reconstruction by 7% in comparison to non-scatter correction (26). Therefore, the future studies should examine the scatter correction methods.

This study was associated with several limitations. First, SPECT images were reconstructed using FBP because it is a standard quantitative method for brain SPECT in Japan. Iterative reconstruction may improve both image quality and quantitative accuracy; consequently, iterative reconstruction methods should be further investigated in the future studies. Second, attenuation correction was performed using Chang’s method since CT-based attenuation correction has not yet become widely available in most of the clinical institutions. Regarding this, the CT-based attenuation correction should be examined in the future studies. Finally, the present investigation was a phantom study; therefore, clinical studies are required in the future.

## Conclusion

As the findings of the current study indicated, the optimal reconstruction parameters differed according to the devices and collimators that were utilized. The fan beam collimator could improve the quantitative accuracy of dopamine transporter SPECT.

## Conflicts of interest

No potential conflicts of interest were disclosed.

## References

[1] Djang DS, Janssen MJ, Bohnen N, Booij J, Henderson TA, Herholz K (2012). SNM practice guideline for dopamine transporter imaging with 123I-ioflupane SPECT 10. J Nucl Med.

[2] Darcourt J, Booji J, Tatsch K, Varrone A, Vander Borght T, Kapucu OL (2010). EANM procedure guidelines for brain neurotransmission SPECT using 123I-labelled dopamine transporter ligands, version 2. Eur J Nucl Med Mol Imaging.

[3] O’Sullivan JD, Lees AJ (2000). Nonparkinsonian tremors. Clin Neuropharmacol.

[4] Furukawa Y, Kish SJ (1999). Dopa-responsive dystonia: recent advances and remaining issues to be addressed. Mov Disord.

[5] Albanece A, Colosimo C, Lees AJ, Tonali P (1996). The clinical diagnosis of multiple system atrophy presenting as pure Parkinsonism. Adv Neurol.

[6] Lavalaye J, Booij J, Reneman L, Habraken JB, van Royen EA (2000). Effect of age and gender on dopamine transporter imaging with [123I]-FP-CIT SPET in healthy volunteers. Eur J Nucl Med Mol Imaging.

[7] Leenders KL, Palmer AJ, Quinn N, Clark JC, Firnau G, Garnett ES (1986). Brain dopamine metabolism in patients with Parkinson’s disease measured with positron emission tomography. J Neurol Neurosurg Psychiatry.

[8] Leenders KL, Salmon EP, Tyrrell P, Perani D, Brooks DJ, Sager H (1990). The nigrostriatal dopaminergic system assessed in vivo by positron emission tomography in healthy volunteer subjects and patients with Parkinson’s disease. Arch Neurol.

[9] Ishikawa T, Dhawan V, Kazumata K, Chaly T, Mandel F, Neumeyer J (1996). Comparative nigrostriatal dopaminergic imaging with iodine-123-beta CIT- FP/SPECT and fluorine-18-FDOPA/PET. J Nucl Med.

[10] Eshuis SA, Maguire RP, Leender KL, Jonkman S, Jager PL (2006). Comparison of FP-CIT SPECT with F-DOPA PET in patients with de novo and advanced Parkinson’s disease. Eur J Nucl Med Mol Imaging.

[11] Eshuis SA, Jager PL, Maguire RP, Jonkman S, Dierckx RA, Leenders KL (2009). Direct comparison of FP-CIT SPECT and F-DOPA PET in patients with Parkinson’s disease and healthy controls. Eur J Nucl Med Mol Imaging.

[12] Maebatake A, Sato M, Kagami R, Yamashita Y, Komiya I, Himuro K (2015). An anthropomorphic phantom study of brain dopamine transporter SPECT images obtained using different SPECT/CT devices and collimators. J Nucl Med Technol.

[13] Maebatake A, Imamura A, Kodera Y, Yamashita Y, Himuro K, Baba S (2016). Evaluation of iterative reconstruction method and attenuation correction in brain dopamine transporter SPECT using an anthropomorphic striatal phantom. Asia Ocean J Nucl Med Biol.

[14] Tsui BM, Gullberg GT, Edgerton ER, Gilland DR, Perry JR, McCartney WH (1986). Design and clinical utility of a fan beam collimator for SPECT imaging of the head. J Nucl Med.

[15] Kouris K, Clarke GA, Jarrit PH, Townsend CE, Thomas SN (1993). Physical performance evaluation of the Toshiba GCA-9300A triple-headed. J Nucl Med.

[16] King MA, Mukherjee JM, Kӧnik A, Zubal IG, Dey J, Licho R (2016). Design of a multi-pinhole collimator for I-123 DaTscan Imaging on dual-headed SPECT systems in combination with a fan-beam collimator. IEEE Trans Nucl Sci.

[17] Iida H, Hori Y, Ishida K, Imabayashi E, Matsuda H, Takahashi M (2013). Three-dimensional brain phantom containing bone and grey matter structures with a realistic head contour. Ann Nucl Med.

[18] Snay ER, Treves ST, Fahey FH (2011). Improved quality of pediatric 123I-MIBG images with medium-energy collimators. J Nucl Med Technol.

[19] Verberne HJ, Feenstra C, de Jong WM, Somsen GA, van Eck-Smit BL, Busemann Sokole E (2005). Influence of collimator choice and simulated clinical conditions on 123I-MIBG heart/mediastinum ratios: a phantom study. Eur J Nucl Med Mol Imaging.

[20] Crespo C, Gallego J, Cot A, Falcón C, Bullich S, Pareto D (2008). Quantification of dopaminergic neurotransmission SPECT studies with 123I-labelled radioligands A comparison between different imaging systems and data acquisition protocols using Monte Carlo simulation. Eur J Nucl Med Mol Imaging.

[21] Frouin V, Comtat C, Reilhac A, Grégoire MC (2002). Correction of partial-volume effect for PET striatal imaging: fast implementation and study of robustness. J Nucl Med.

[22] Soret M, Koulibaly PM, Darcourt J, Hapdey S, Buvat I (2003). Quantitative accuracy of dopaminergic neurotransmission imaging with 123I SPECT. J Nucl Med.

[23] Buvat I, Soret M, Hapdey S, Riddell C, Benali H, Di Paola R (2000). Respective importance of scatter, attenuation, collimator response and partial volume effect corrections for accurate quantification in 123I dopamine receptor imaging. IEEE Med Imaging Conf Record.

[24] Soret M, Koulibaly PM, Darcourt J, Buvat I (2006). Partial volume effect correction in SPECT for striatal uptake measurements in patients with neurodegenerative disease: impact upon patient classification. Eur J Nucl Med Mol Imaging.

[25] Tossici-Bolt L, Hoffman SM, Kemp PM, Mehta RL, Fleming JS (2006). Quantification of [123I]FP-CIT SPECT brain images: an accurate technique for measurement of the specific binding ratio. Eur J Nucl Med Mol Imaging.

[26] Cot A, Falcón C, Crespo C, Sempau J, Pareto D, Bullich S (2005). Absolute quantification in dopaminergic neurotransmission SPECT using a Monte Carlo-based scatter correction and fully 3-dimensional reconstruction. J Nucl Med.

